# Soluble Markers of the Toll-Like Receptor 4 Pathway Differentiate between Active and Latent Tuberculosis and Are Associated with Treatment Responses

**DOI:** 10.1371/journal.pone.0069896

**Published:** 2013-07-16

**Authors:** Siri L. Feruglio, Marius Trøseid, Jan Kristian Damås, Dag Kvale, Anne Ma Dyrhol-Riise

**Affiliations:** 1 Institute of Clinical Medicine, Faculty of Medicine, University of Oslo, Oslo, Norway; 2 Department of Infectious Diseases, Oslo University Hospital, Oslo, Norway; 3 Department of Infectious Diseases, St. Olavs Hospital, Trondheim, Norway; 4 Institute of Cancer Research and Molecular Medicine, Norwegian University of Science and Technology, Trondheim, Norway; 5 Department of Clinical Science, University of Bergen, Bergen, Norway; National Institute for Infectious Diseases (L. Spallanzani), Italy

## Abstract

**Background:**

Biomarkers to differentiate between active tuberculosis (TB) and latent TB infection (LTBI) and to monitor treatment responses are requested to complement TB diagnostics and control, particularly in patients with multi-drug resistant TB. We have studied soluble markers of the Toll-like-receptor 4 (TLR-4) pathway in various stages of TB disease and during anti-TB treatment.

**Methods:**

Plasma samples from patients with culture confirmed drug-sensitive TB (n = 19) were collected before and after 2, 8 and 24 weeks of efficient anti-TB treatment and in a LTBI group (n = 6). Soluble (s) CD14 and myeloid differentiation-2 (MD-2) were analyzed by the Enzyme-linked immunosorbent assay (ELISA). Lipopolysaccharide (LPS) was analyzed by the Limulus Amebocyte Lysate colorimetric assay. Nonparametric statistics were applied.

**Results:**

Plasma levels of sCD14 (p<0.001), MD-2 (p = 0.036) and LPS (p = 0.069) were elevated at baseline in patients with untreated active TB compared to the LTBI group. MD-2 concentrations decreased after 2 weeks of treatment (p = 0.011), while LPS levels decreased after 8 weeks (p = 0.005). In contrast, sCD14 levels increased after 2 weeks (p = 0.047) with a subsequent modest decrease throughout the treatment period. There was no significant difference in concentrations of any of these markers between patients with pulmonary and extrapulmonary TB or between patients with or without symptoms.

**Conclusion:**

Our data suggest that plasma levels of LPS, MD-2 and sCD14 can discriminate between active TB and LTBI. A decline in LPS and MD-2 concentrations was associated with response to anti-TB treatment. The clinical potential of these soluble TLR-4 pathway proteins needs to be further explored.

## Introduction

Tuberculosis (TB) is a global challenge and there were an estimated 8.7 million incident cases in 2011, 13% were co-infected with human immunodeficiency virus (HIV) and 20% of previously treated cases had multi-drug resistant (MDR)-TB [1]. Both TB culture, which is the diagnostic gold standard, and the *Xpert MTB/RIF assay* often fail in cases of extrapulmonary TB, in HIV-infection and in children [2] and TB diagnosis is often made on clinical criteria. Thus, leading stakeholders have called for intensified basic scientific research to explore TB immunopathogenesis and screen for diagnostic and prognostic biomarker candidates that could complement diagnosis [3]–[5]. Predictors of treatment efficacy are particularly relevant in MDR-TB with less efficient therapeutic regimens of longer duration.

A number of TB antigens have been tested as potential biomarkers [6], [7]. Available serology tests are unreliable even though they are in extensive use in some TB high-endemic countries [8], [9]. Several meta-analyses show that the cellular interferon- gamma- release- assays (IGRAs) are at least as sensitive and more specific than the tuberculin skin test (TST) [10]–[13]. Still, they do not discriminate between latent TB infection (LTBI) and active TB and cannot monitor therapy.

*Mycobacterium tuberculosis* (*Mtb*) possesses a unique cell envelope with an outer lipid layer of mycolic acids and intercalated additional complex lipids, which have been the targets for immunogenic, diagnostic and drug research [14]. *Mtb* synthesize two unique families of cytoplasmic polymethylated polysaccharides, the methylglucose lipopolysaccharides (MGLPs) and the methylmannose polysaccharides (MMPs) [15]–[17]. Cell wall-associated lipoglycans such as lipoarabinomannan (LAM) inhibit the expression of pro-inflammatory cytokines by activated macrophages and has been identified as a major virulence factor of *Mtb* [18]. Whereas the point-of-care LAM urine antigen test could aid in the diagnosis of advanced HIV-associated TB [19], the overall low sensitivity in HIV negative TB patients may still limit this test’s utility [20].

Toll-like receptors (TLR), essential membrane receptors that recognize pathogen-associated molecules, interact with specific *Mtb* structures triggering signaling pathways [21]. Various types of LPS induce synthesis of proinflammatory cytokines through binding to the TLR-4/myeloid differentiation-2 (MD-2) complex [22], [23]. Several soluble proteins regulate the signaling of LPS through the TLR-4 pathway. CD14, a glycoprotein expressed on the surface of macrophages, monocytes and phagocytes seems to be essential in the host response against microbial infection [24], [25]. Soluble (s) CD14 serves as a receptor for the complex of LPS and LPS binding protein (LBP) and plays a central role by transferring LPS to MD-2, thus activating immune responses to bacterial endotoxins [22], [26]. Elevated levels of sCD14 are associated with various systemic inflammatory diseases including TB [27], and increased levels of MD-2 have been detected in plasma from patients with severe sepsis as well as in HIV infection [28], [29]. It has been shown that during anti-TB treatment the levels of sCD14 slowly decline [27] and the levels of LBP are lower in treated compared to untreated TB patients [30]. To our knowledge, however, there are no longitudinal studies of circulating levels of MD-2, sCD14 and LPS in TB patients or of the possible effects of anti-TB treatment upon these potential biomarkers. Our pilot study indicates that plasma levels of LPS and MD-2 can discriminate between various stages of TB disease and possibly predict responses to therapy.

## Materials and Methods

### Study Participants

Nineteen patients with active culture-confirmed TB, ten with pulmonary and nine with extrapulmonary TB, were recruited from the Department of Infectious Diseases, Oslo University Hospital, Norway. Demographical and clinical data were as described in Table 1. Two patients were native Norwegians (age 81 and 92) and the remaining were immigrants, predominately from Africa (n = 9) and Asia (n = 6). Patients were classified as symptomatic when they presented with two or more of the following symptoms: fever (temperature>38°C), weight loss, wasting, cough and night-sweat. Nine had symptomatic TB while the others were diagnosed in routine screening or investigations for other medical conditions. The erythrocyte sedimentation rate (ESR), C-reactive protein (CRP), thoracic X ray, acid-fast staining and culture of sputum results were registered upon inclusion. All *Mtb* strains were drug-sensitive. Patients were treated with a standard combination of four drugs (isoniazid, rifampicin, ethambutol and pyrazinamide) for 2 months, followed by isoniazid and rifampicin for 4 months. Sputum samples were assessed after 2 and 6 months of treatment and patients were also evaluated after one year according to clinical practice. All patients responded to treatment, pulmonary TB patients with sputum conversion and culture clearing whereas extrapulmonary TB patients demonstrated clinical improvement and normalization of inflammation parameters (CRP, ESR). No relapse was observed during the year of follow-up. Six subjects with LTBI were recruited from Haukeland University Hospital, Bergen. Two were native Norwegians and four immigrants. All LTBI tested positive with the QuantiFERON-TB® test (Cellestis/Qiagen). Thoracic X-ray, sputum smears and clinical examinations were performed and there were no signs of active TB in any of the persons. Both active TB and LTBI patients were HIV negative.

**Table 1:** Demographic and clinical characteristics of study participants.

		Active TB	Latent TB
		N = 19	N = 6
**Median age (years)** 1		32 (26–41)	40 (26–54)
**Sex**	Female	9	4
	Male	10	2
**Country of origin**	Africa	9	2
	Asia	6	2
	Europe	4^2^	2^3^
**Localisation of disease**	Pulmonary	10	
	Extrapulmonary	9	
**Symptomatic TB** 4		9	

1 Interquartile range in brackets.

2 Including two native Norwegians.

3 All native Norwegians.

4 ≥2 of the following symptoms: fever (temperature>38°C), weight loss, wasting, cough and night-sweat.

Blood samples were obtained by venipuncture prior to start of treatment (baseline), after 2, 8 and 24 weeks in the active TB group and at the time of diagnosis in the LTBI group. Plasma samples were separated and snap-frozen within 30 minutes and stored at minus 80°C until analysis.

The study was approved by the respective Regional Boards for Medical and Health Research Ethics in Norway (in Health region South-East and West). Written informed consent was obtained from all participants.

### Lipopolysaccharide Assay

**Figure 1:**
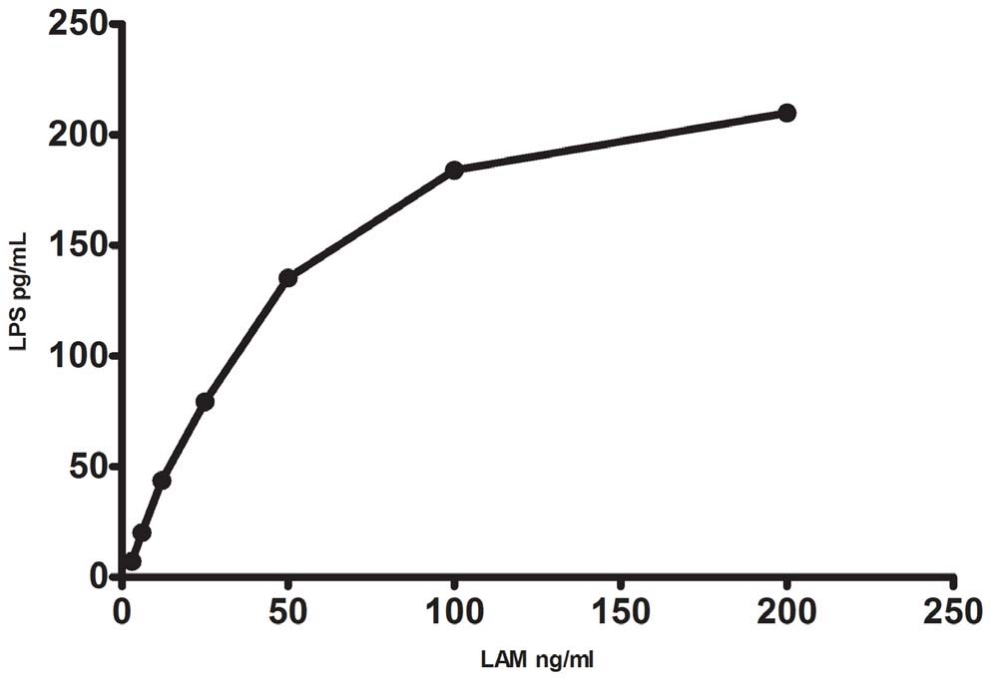
Lipoarabinomannan (LAM) detected by the Lipopolysaccaride (LPS) assay. Relation between Lipoarabinomannan (LAM) standard concentrations from the Enzyme-linked immunosorbent assay (ELISA) kit and Lipopolysaccaride (LPS) concentrations analysed by the Limulus Amebocyte Lysate colorimetric assay (LAL).

We observed that the Limulus Amebocyte Lysate colorimetric assay (LAL) (Lonza, Walkersville, MD, USA) could detect TB-specific LAM from the General LAM Enzyme-linked immunosorbent assay (ELISA) kit (Wuhan Eiaab Science, Whuan, China) in a dose dependent manner, indicating that the LAL assay could detect mycobacterial LPS (Fig. 1). We then analyzed LAM in plasma samples from the active TB group in 8- and 24-fould dilutions according to the manufacturer’s instructions. However, about 40% of the tested samples were below twice the background optic density value and thereby not quantifiable (data not shown).

LPS concentrations in plasma were then analyzed in duplicates by the LAL colorimetric assay according to the manufacturer’s instructions with the following modifications: plasma samples were diluted 10-fold to avoid interference with background color and preheated to 68°C for 12 minutes prior to analyses to dissolve immune complexes as previously described [29]. Test samples were mixed with the LAL supplied in the test kit and incubated at 37°C for 10 minutes. A substrate solution was then mixed with the LAL sample and incubated at 37°C for an additional 6 minutes and the reaction was stopped. A conversion to yellow developed if LPS was present. The absorbance of the sample was determined by spectrophotometer and concentration calculated from a standard curve.

### Myeloid Differentiation-2 ELISA

MD-2 was analyzed by an in-house ELISA as previously described [31]. Briefly, MD-2 was captured on immunoplates coated with a TLR-4-Fc fusion protein and detected with digoxygenin labeled MD-2 mAb 5D7 or digoxigenin labeled IIC1 and anti-digoxygenin-HRP (Roche). The MD-2 standard was purchased from R&D Systems.

### Soluble CD14 ELISA

sCD14 was analyzed by a sandwich ELISA coated with CD14-specific monoclonal antibodies according to the manufacturer’s instructions (R&D, Minneapolis, MN, USA). Samples were diluted 300-fold and incubated in duplicates for three hours at room temperature. After a total of four washing steps, the wells were incubated with sCD14 conjugate solution for one hour. The cells were washed, substrate solution added, incubated for 30 minutes and stopped. A conversion to blue developed if sCD14 was present, the absorbance of the sample was determined by spectrophotometer and concentrations were calculated from a standard curve.

### Inflammation Markers

CRP was estimated by Immuno Turbidometric assay (Cobas 8000, Roche Diagnostics, USA) and ESR according to Westergren Method (StaRRsed Compact Automatic Analyzer, RRmechatronic, Hoom, the Netherlands).

### Statistical Analysis

Statistical analyses were performed by Statistica v 7.0 (Statsoft, Tulsa, OK, USA). Nonparametrical statistical methods were applied for group-wise comparison. Mann-Whitney U test and two-tailed Wilcoxon matched pairs test for dependent variables. Correlation analyses were performed using the Spearman method. A significance level of 0.05 was used. All values are presented as median and interquartile range (IQR).

## Results

### Elevated Plasma Lipopolysaccharide Levels Decline during TB Treatment

**Figure 2:**
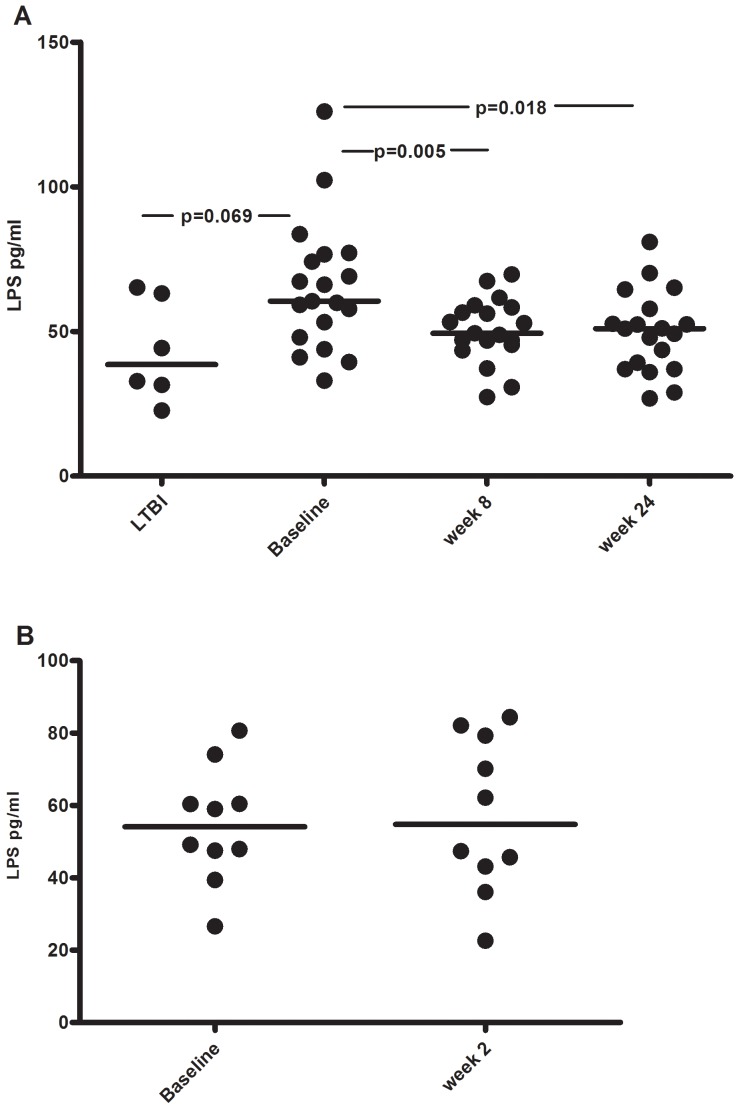
Lipopolysaccharide levels at various stages of TB infection and during therapy. Lipopolysaccharide (LPS) concentrations (pg/ml) in plasma from A) patients with LTBI (n = 6) and active TB (n = 19) at baseline and after 8 and 24 weeks of anti-TB therapy and B) active TB (n = 10) at baseline and after 2 weeks of anti-TB therapy. Horizontal lines represent the median values. Significant p values (<.05) between groups and time-points are indicated.

Plasma levels of LPS at baseline were higher, although not significant, in the active TB group compared to the LTBI group (60 pg/mL [44–77] vs. 39 pg/mL [31–63], p = 0.069) (Fig. 2A). In the active TB group LPS levels decreased significantly during treatment (49 pg/mL [43–57], p = 0.005 at week 8). The decline in LPS level was maintained at week 24 (48 pg/mL [37–52], p = 0.018). No significant decline was observed from baseline to week 2, measured in ten of the patients (Fig. 2B). LPS concentrations did not differ significantly between patients with pulmonary and extrapulmonary TB (60 pg/mL [44–69] vs. 59 pg/mL [48–77]).

### Elevated Plasma Myeloid Differentiation-2 Levels Rapidly Decline during TB Treatment

**Figure 3:**
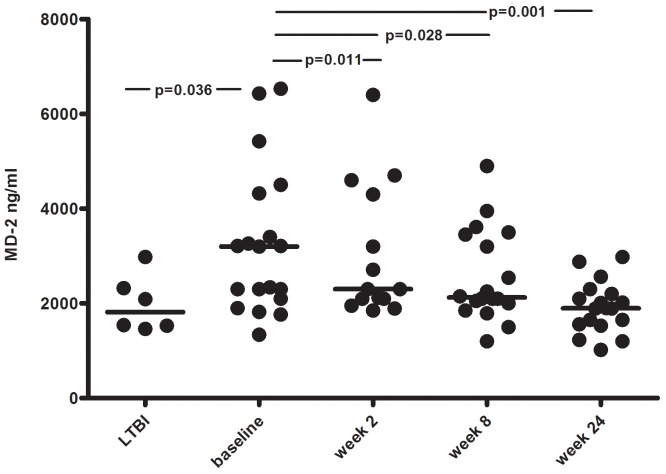
Myeloid differentiation-2 levels at various stages of TB infection and during therapy. Myeloid differentiation-2 (MD-2) concentrations (ng/ml) in plasma from patients with LTBI (n = 6) and active TB (n = 19) at baseline and after 2, 8 and 24 weeks of anti-TB therapy. Horizontal lines represent the median values. Significant p values (<.05) between groups and time-points are indicated.

Plasma levels of MD-2 at baseline were higher in the active TB group compared to the LTBI group (3200 ng/ml [2100–4320]) vs. 1815 ng/ml [1530–2320], p = 0.036) (Fig. 3). In active TB patients there was a rapid decline in the level of MD-2 compared to baseline, significant already after 2 weeks of treatment (2300 ng/ml [2100–4300], p = 0.011), and still significant at week 8 (2125 ng/ml [2000–3450], p = 0.028) and week 24 (1895 ng/ml [1560–2200], (p = 0.001). MD-2 concentrations did not differ significantly between patients with pulmonary and extrapulmonary TB (2655 ng/mL [1820–5420] vs. 3200 ng/ml [2300–3260]).

### Elevated Plasma Levels of sCD14 during TB Treatment

**Figure 4:**
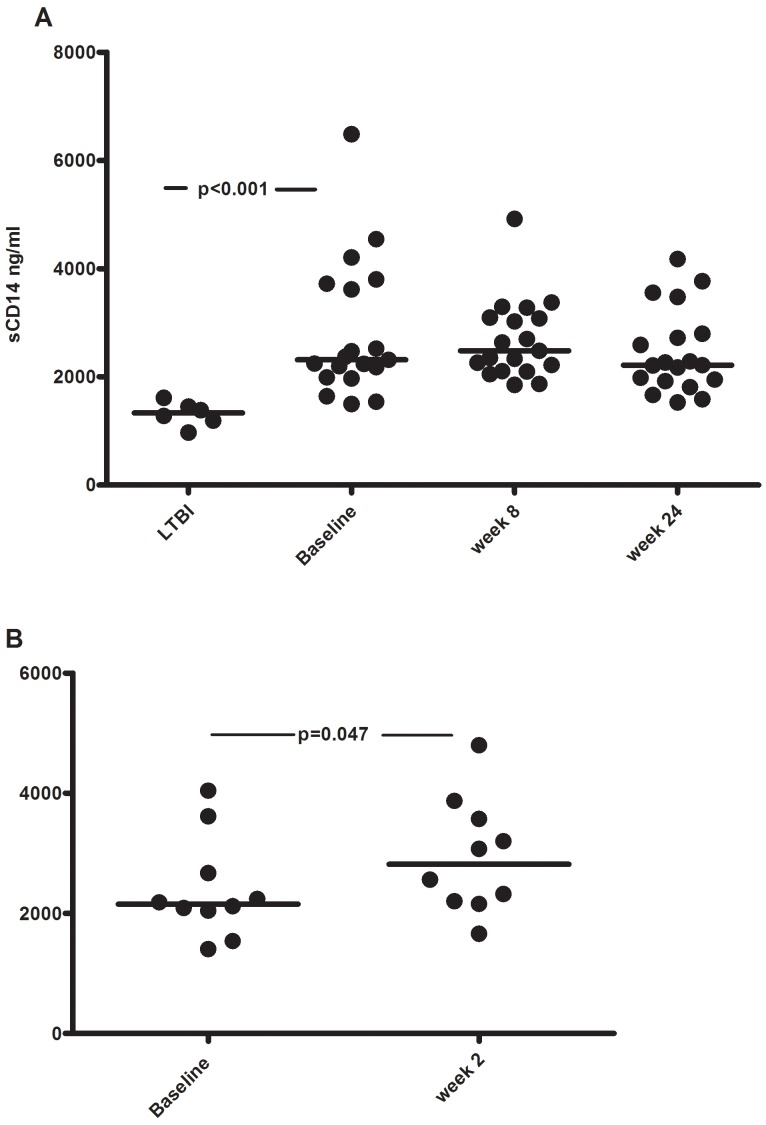
sCD14 levels at various stages of TB infection and during therapy. Soluble CD14 concentrations (ng/ml) in plasma from A) patients with LTBI (n = 6) and active TB (n = 19) at baseline and after 8 and 24 weeks of anti-TB therapy and B) active TB (n = 10) at baseline and after 2 weeks of anti-TB therapy. Horizontal lines represent the median values. Significant p values (<.05) between groups and time-points are indicated.

Plasma levels of sCD14 at baseline were higher in the active TB group compared to the LTBI group (2320 ng/ml [1990–3720] vs. 1335 ng/ml [1190–1450], p<0.001) (Fig. 4A). After 2 weeks of therapy there was a significant increase in sCD14 in the active TB group (2820 ng/ml [2190–3650], p = 0.047) (Fig. 4B). A slightly decrease in median sCD14 was observed after 24 weeks of therapy (2220 ng/ml [1920–2800]), although not significant (p = 0.064) (Fig. 4A). sCD14 levels did not differ between patients with pulmonary and extrapulmonary TB (2450 ng/ml [2200–3720] vs. 2240 ng/mL [1640–2475]).

### Correlations between sCD14, LPS, MD-2 and Inflammation Markers

**Figure 5:**
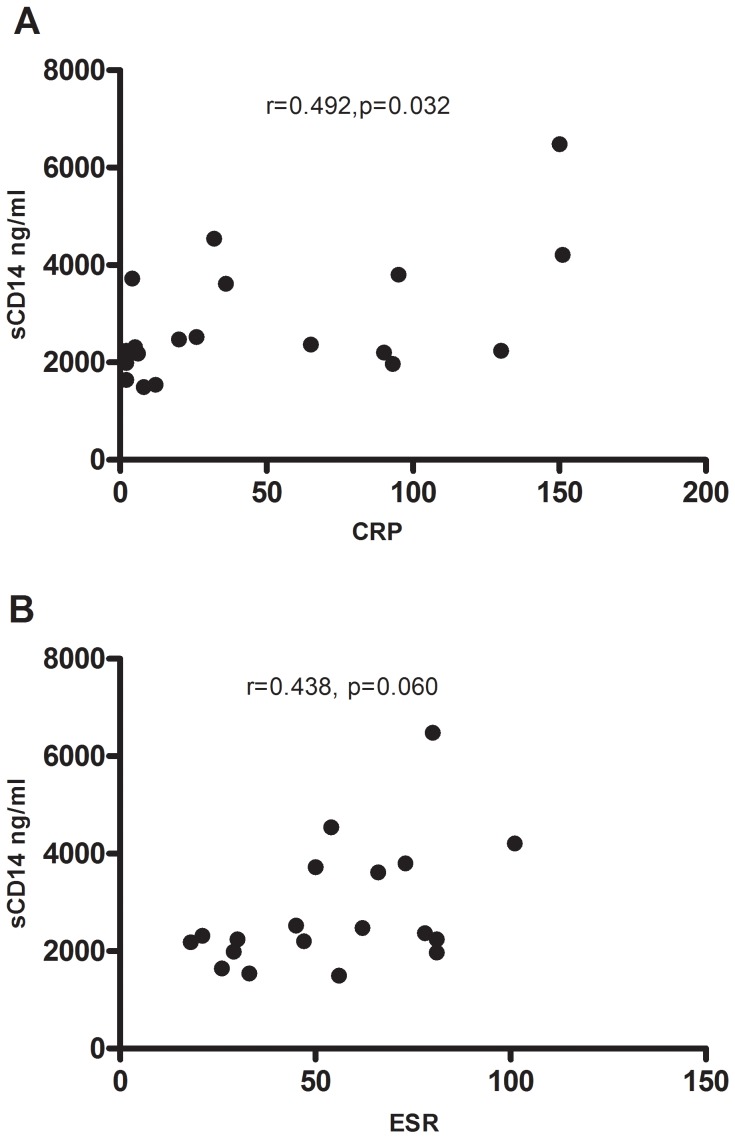
Correlations between sCD14 and inflammation markers. Correlations between inflammation markers in the active TB group (n = 19) between A) CRP and sCD14 (r = 0.492, p = 0.032), B) ESR and sCD14 (r = 0.438, p = 0.060).

Nine active TB patients were categorized as symptomatic at baseline. The inflammation markers CRP and ESR were significantly higher in the symptomatic compared to the asymptomatic TB group (77 mg/L [36–95] vs. 24 mg/L [3]–[18], p = 0.017 and 67 mm [50–80] vs. 43 mm [27–56], p = 0.028, respectively), but CRP and ESR could not discriminate between pulmonary and extrapulmonary TB. At baseline there were significant correlations between CRP and sCD14 (r = 0.492, p = 0.032) while the correlation between ESR and sCD14 was modest (r = 0.438, p = 0.060) (Fig. 5). LPS or MD-2 did not correlate to either CRP or ESR. There were no significant differences in levels of LPS, sCD14 and MD-2 between patients with high or low symptom score or between patients with pulmonary and extrapulmonary TB (data not shown).

## Discussion

Mycobacterial cell wall components are immunogenic and thus potential biomarkers for TB [14]. Proteins involved in the regulation of innate immunity through the TLR-4 pathway could also serve as surrogates for TB disease [21]. We have analyzed plasma concentrations of LPS, sCD14 and MD-2 in patients with various clinical manifestations and stages of TB infection and during anti-TB treatment. We have shown that plasma levels of MD-2, sCD14 and possibly LPS are higher in patients with active TB than in LTBI. Most important, we found that the decline in LPS and MD-2 concentrations was associated with response to anti-TB treatment. sCD14 transfers LPS to MD-2, thus activating immune responses to bacterial endotoxins [22], [26]. Immunoregulatory cytokines such as IL-10 downregulate protective immune responses in chronic active TB infection [32] and seems to be a driving force for increased release of MD-2 and CD14 during systemic inflammation [31]. Thus, the decline in these markers, for MD-2 measured already after 2 weeks, could reflect an early down-regulation of immune activation during effective anti-TB therapy**.** The decline in LPS levels supports an Indian study where they isolated an immunodominant LPS antigen from Mtb H37Rv and compared LPS with three commercially available *Mtb*-specific antigens for detection of TB [33]. They found that LPS sensitivity was 84% and specificity 97%, suggesting that the LPS antigen could be used in sero-diagnosis of TB. Further, a recent study on circulating biomarkers demonstrated that LPS was elevated in plasma from children with pulmonary TB, arguing that LPS might serve as a marker of innate immune activation [34]. Previous studies have also shown that proteins involved in the innate immune activation are elevated in other infectious diseases as parasitic infections [35], HIV infection [36] and hepatitis [37]. Increased circulating LPS could result from chronic immune activation caused by microbial translocation as has been demonstrated in HIV infection [36], [38]. We think, however, that in our patients this is less likely as none had gastrointestinal or disseminated TB. In contrast to that found for MD-2 we did not record any changes in LPS levels after 2 weeks of treatment. Wood *et al* showed a decline in urine LAM concentrations in South-African patients treated for active TB between 2 and 8 weeks of treatment [39], and given the apparently higher sensitivity of our LAL-assay, further investigations of the time kinetics of LPS during anti-TB should be performed.

We found higher levels of sCD14 in plasma from patients with active TB compared to those with LTBI, as shown in previous studies [27], [30] **.** We observed a very modest and not statistical significant decline of sCD14 throughout the 24 week observation period. Whereas Juffermans *et al* found normalization of sCD14 levels in TB patients that had completed curative treatment [30], Lawn *et al* showed that the levels of sCD14 declined slowly and were still significantly elevated after 3 months of treatment, in support of our data [27].

Still, we detected increased plasma levels of sCD14 at week 2 of TB treatment in a smaller group of patients where plasma samples were available. Lawn *et al* also observed a similar transient increase during the first month of treatment, followed by a slow decline in serum sCD14 concentrations [27]. They argue that the initial rise in sCD14 levels might represent the host response to the increase of mycobacterial antigen load due to therapy correspondingly to what has been previously shown for tumour necrosis factor alpha (TNF-α) [40].Our data supports this conclusion, although the mechanisms need to be further documented by *in vitro* studies.

In our study sCD14 levels correlated to some extent to CRP levels, suggesting that both these markers reflect the level of inflammation [41]. In contrast, we did not find correlations between inflammation markers and LPS or MD-2 plasma levels, respectively. It has been shown that cell wall-associated lipids inhibit LPS-induced TNF-α release by a sCD14-dependent pathway, while IL-12 p40 inhibition is sCD14-independent [42]. Such mechanisms could explain the diverse associations detected for the various markers.

In our study the parameters did not discriminate between either disease localization or symptom score. One would assume significantly lower plasma levels of sCD14 in extrapulmonary TB compared to pulmonary TB due to the expected various levels of bacillary load [30]. However, all extrapulmonary TB patients in our study had positive TB cultures and neither CRP nor ESR could discriminate between pulmonary and extrapulmonary TB. In fact, several of the extrapulmonary cases had much higher levels of inflammation markers than that found in many of the patients with pulmonary TB. This, as well as the relatively low numbers of patients in each group, could explain why we did not find any significant difference for sCD14, LPS and MD-2 between these two patient groups. Of note, the rapid decline in MD-2 in response to therapy was also seen in those patients with none or few symptoms where clinical improvement would be of less use as parameter of therapy efficacy. Thus, MD-2 could potentially serve as a complimentary biomarker when evaluating the initial response to therapy in patients with few symptoms.

The limitation of this study is the small sample size, which increases the risk of type II statistical errors. However, since type I errors are less likely, both our findings that MD-2 and sCD14 can discriminate between latent and active TB and that MD-2 and LPS are associated with treatment responses are probably valid. Further, the LAL assay might be less specific in patients with concurrent HIV infection and increased levels of LPS due to microbial translocation [43].

In conclusion, based on our data and other reports, a multi-parameter approach including biomarkers must be considered to aid in TB diagnosis and monitor efficacy of TB treatment [5], [44]. The clinical importance of these TLR-4 regulatory proteins is yet to be determined. Still, our explorative novel data indicate that these soluble markers, in particular MD-2, need to be further studied in larger cohorts of TB patients.

## References

[1] World Health Organization (2010) WHO, Global tuberculosis report. Available: http://www.who.int/tb/publications/global_report/en/.

[2] Lawn SD, Mwaba P, Bates M, Piatek A, Alexander H, et al (2013) Advances in tuberculosis diagnostics: the Xpert MTB/RIF assay and future prospects for a point-of-care test. Lancet Infect Dis 13: 349–361. 23531388 10.1016/S1473-3099(13)70008-2 PMC4844338

[3] Phillips PP, Davies GR, Mitchison DA (2010) Biomarkers for tuberculosis disease activity, cure, and relapse. Lancet Infect Dis 10: 69–70. 20113973 10.1016/S1473-3099(09)70256-7

[4] Ottenhoff TH, Ellner JJ, Kaufmann SH (2012) Ten challenges for TB biomarkers. Tuberculosis (Edinb ) 92 Suppl 1S17–20. 22441153 10.1016/S1472-9792(12)70007-0

[5] Parida SK, Kaufmann SH (2010) The quest for biomarkers in tuberculosis. Drug Discov Today 15: 148–157. 19854295 10.1016/j.drudis.2009.10.005

[6] Chegou NN, Essone PN, Loxton AG, Stanley K, Black GF, et al (2012) Potential of host markers produced by infection phase-dependent antigen-stimulated cells for the diagnosis of tuberculosis in a highly endemic area. PLoS One 7(6): e38501 doi:10.1371/journal.pone.0038501. Epub 2012 Jun 5 22693640 10.1371/journal.pone.0038501 PMC3367928

[7] Chegou NN, Black GF, Loxton AG, Stanley K, Essone PN, et al (2012) Potential of novel Mycobacterium tuberculosis infection phase-dependent antigens in the diagnosis of TB disease in a high burden setting. BMC Infect Dis 12: 10. 22260319 10.1186/1471-2334-12-10 PMC3282638

[8] WHO (2010) Commercial Serodiagnostic Tests for Diagnosis of Tuberculosis. Policy Statement. Available: http://whqlibdoc.who.int/publications/2011/9789241502054_eng.pdf. 26158189

[9] Steingart KR, Henry M, Laal S, Hopewell PC, Ramsay A, et al (2007) Commercial serological antibody detection tests for the diagnosis of pulmonary tuberculosis: a systematic review. PLoS Med. Jun 4(6): e202. 10.1371/journal.pmed.0040202 PMC1891320 17564490

[10] Dyrhol-Riise AM, Gran G, Wentzel-Larsen T, Blomberg B, Haanshuus CG, et al (2010) Diagnosis and follow-up of treatment of latent tuberculosis; the utility of the QuantiFERON-TB Gold In-tube assay in outpatients from a tuberculosis low-endemic country. BMC Infect Dis 10: 57. 20210999 10.1186/1471-2334-10-57 PMC2842274

[11] Rangaka MX, Wilkinson KA, Glynn JR, Ling D, Menzies D, et al (2012) Predictive value of interferon-gamma release assays for incident active tuberculosis: a systematic review and meta-analysis. Lancet Infect Dis 12: 45–55. 21846592 10.1016/S1473-3099(11)70210-9 PMC3568693

[12] Nienhaus A, Schablon A, Costa JT, Diel R (2011) Systematic review of cost and cost-effectiveness of different TB-screening strategies. BMC Health Serv Res 11: 247. 21961888 10.1186/1472-6963-11-247 PMC3196701

[13] Pai M, Zwerling A, Menzies D (2008) Systematic review: T-cell-based assays for the diagnosis of latent tuberculosis infection: an update. Ann Intern Med 149: 177–184. 18593687 10.7326/0003-4819-149-3-200808050-00241 PMC2951987

[14] Mishra AK, Driessen NN, Appelmelk BJ, Besra GS (2011) Lipoarabinomannan and related glycoconjugates: structure, biogenesis and role in Mycobacterium tuberculosis physiology and host-pathogen interaction. FEMS Microbiol Rev 35: 1126–1157. 21521247 10.1111/j.1574-6976.2011.00276.x PMC3229680

[15] Mendes V, Maranha A, Alarico S, Empadinhas N (2012) Biosynthesis of mycobacterial methylglucose lipopolysaccharides. Nat Prod Rep 29: 834–844. 22678749 10.1039/c2np20014g

[16] Jackson M, Brennan PJ (2009) Polymethylated polysaccharides from Mycobacterium species revisited. J Biol Chem 284: 1949–1953. 18786916 10.1074/jbc.R800047200 PMC2629103

[17] Kaur D, Pham H, Larrouy-Maumus G, Riviere M, Vissa V, et al (2009) Initiation of methylglucose lipopolysaccharide biosynthesis in mycobacteria. PLoS One. 4(5): e5447 doi:10.1371/journal.pone.0005447. Epub 2009 May 7 10.1371/journal.pone.0005447 PMC2674218 19421329

[18] Briken V, Porcelli SA, Besra GS, Kremer L (2004) Mycobacterial lipoarabinomannan and related lipoglycans: from biogenesis to modulation of the immune response. Mol Microbiol 53: 391–403. 15228522 10.1111/j.1365-2958.2004.04183.x

[19] Lawn SD, Kerkhoff AD, Vogt M, Wood R (2012) Diagnostic accuracy of a low-cost, urine antigen, point-of-care screening assay for HIV-associated pulmonary tuberculosis before antiretroviral therapy: a descriptive study. Lancet Infect Dis 12: 201–209. 22015305 10.1016/S1473-3099(11)70251-1 PMC3315025

[20] Swaminathan S, Rekha VV (2012) Antigen detection as a point-of-care test for TB: the case of lipoarabinomannan. Future Microbiol 7: 559–564. 22568711 10.2217/fmb.12.34

[21] Kleinnijenhuis J, Oosting M, Joosten LA, Netea MG, Van CR (2011) Innate immune recognition of Mycobacterium tuberculosis. Clin Dev Immunol. 2011: 405310 doi:10.1155/2011/405310. Epub 2011 Apr 7 10.1155/2011/405310 PMC3095423 21603213

[22] Palsson-McDermott EM, O’Neill LA (2004) Signal transduction by the lipopolysaccharide receptor, Toll-like receptor-4. Immunology 113: 153–162. 15379975 10.1111/j.1365-2567.2004.01976.x PMC1782563

[23] da Silva CJ, Ulevitch RJ (2002) MD-2 and TLR4 N-linked glycosylations are important for a functional lipopolysaccharide receptor. J Biol Chem 277: 1845–1854. 11706042 10.1074/jbc.M109910200

[24] Kirkland TN, Viriyakosol S (1998) Structure-function analysis of soluble and membrane-bound CD14. Prog Clin Biol Res 397: 79–87. 9575549

[25] Ziegler-Heitbrock HW, Ulevitch RJ (1993) CD14: cell surface receptor and differentiation marker. Immunol Today 14: 121–125. 7682078 10.1016/0167-5699(93)90212-4

[26] Miyake K (2006) Roles for accessory molecules in microbial recognition by Toll-like receptors. J Endotoxin Res 12: 195–204. 16953972 10.1179/096805106X118807

[27] Lawn SD, Labeta MO, Arias M, Acheampong JW, Griffin GE (2000) Elevated serum concentrations of soluble CD14 in HIV- and HIV+ patients with tuberculosis in Africa: prolonged elevation during anti-tuberculosis treatment. Clin Exp Immunol 120: 483–487. 10844527 10.1046/j.1365-2249.2000.01246.x PMC1905566

[28] Pugin J, Stern-Voeffray S, Daubeuf B, Matthay MA, Elson G, et al (2004) Soluble MD-2 activity in plasma from patients with severe sepsis and septic shock. Blood 104: 4071–4079. 15328161 10.1182/blood-2003-04-1290

[29] Troseid M, Lind A, Nowak P, Barqasho B, Heger B, et al (2013) Circulating levels of HMGB1 are correlated strongly with MD2 in HIV-infection: Possible implication for TLR4-signalling and chronic immune activation. Innate Immunity. 19: 290–297. 10.1177/1753425912461042 23070967

[30] Juffermans NP, Verbon A, van Deventer SJ, Buurman WA, van DH, et al (1998) Serum concentrations of lipopolysaccharide activity-modulating proteins during tuberculosis. J Infect Dis 178: 1839–1842. 9815247 10.1086/314492

[31] Sandanger O, Ryan L, Bohnhorst J, Iversen AC, Husebye H, et al (2009) IL-10 enhances MD-2 and CD14 expression in monocytes and the proteins are increased and correlated in HIV-infected patients. J Immunol 182: 588–595. 19109192 10.4049/jimmunol.182.1.588

[32] Redford PS, Murray PJ, O’Garra A (2011) The role of IL-10 in immune regulation during M. tuberculosis infection. Mucosal Immunol 4: 261–270. 21451501 10.1038/mi.2011.7

[33] Meena LS, Goel S, Sharma SK, Jain NK, Banavaliker JN, et al (2002) Comparative study of three different mycobacterial antigens with a novel lipopolysaccharide antigen for the serodiagnosis of tuberculosis. J Clin Lab Anal 16: 151–155. 11968053 10.1002/jcla.10031 PMC6807707

[34] Pavan KN, Anuradha R, Suresh N, Ganesh R, Shankar J, et al (2013) Circulating biomarkers of pulmonary and extra-pulmonary tuberculosis in children. Clin Vaccine Immunol. 20(5): 704–11. 10.1128/CVI.00038-13 PMC3647760 23486418

[35] Anuradha R, George PJ, Pavan KN, Fay MP, Kumaraswami V, et al (2012) Circulating microbial products and acute phase proteins as markers of pathogenesis in lymphatic filarial disease. PLoS Pathog. 2012 8(6): e1002749 doi:10.1371/journal.ppat.1002749. Epub 2012 Jun 10.1371/journal.ppat.1002749 PMC3369944 22685406

[36] Brenchley JM, Price DA, Schacker TW, Asher TE, Silvestri G, et al (2006) Microbial translocation is a cause of systemic immune activation in chronic HIV infection. Nat Med 12: 1365–1371. 17115046 10.1038/nm1511

[37] Sandler NG, Koh C, Roque A, Eccleston JL, Siegel RB, et al (2011) Host response to translocated microbial products predicts outcomes of patients with HBV or HCV infection. Gastroenterology 141 (4): 1220–30. 10.1053/j.gastro.2011.06.063 PMC3186837 21726511

[38] Troseid M, Nowak P, Nystrom J, Lindkvist A, Abdurahman S, et al (2010) Elevated plasma levels of lipopolysaccharide and high mobility group box-1 protein are associated with high viral load in HIV-1 infection: reduction by 2-year antiretroviral therapy. AIDS 24: 1733–1737. 20502315 10.1097/QAD.0b013e32833b254d

[39] Wood R, Racow K, Bekker LG, Middelkoop K, Vogt M, et al (2012) Lipoarabinomannan in urine during tuberculosis treatment: association with host and pathogen factors and mycobacteriuria. BMC Infect Dis 12: 47. 22369353 10.1186/1471-2334-12-47 PMC3349560

[40] Bekker LG, Maartens G, Steyn L, Kaplan G (1998) Selective increase in plasma tumor necrosis factor alpha and concomitant clinical deterioration after initiating therapy in patients with severe tuberculosis. J Infect Dis 178: 580±4. 10.1086/517479 9697749

[41] Lawn SD, Obeng J, Acheampong JW, Griffin GE (2000) Resolution of the acute-phase response in West African patients receiving treatment for pulmonary tuberculosis. Int J Tuberc Lung Dis 4: 340–344. 10777083

[42] Court N, Rose S, Bourigault ML, Front S, Martin OR, et al (2011) Mycobacterial PIMs inhibit host inflammatory responses through CD14-dependent and CD14-independent mechanisms. PLoS One. 2011 6(9): e24631 doi:10.1371/journal.pone.0024631. Epub 2011 Sep 16 10.1371/journal.pone.0024631 PMC3174970 21949737

[43] Brenchley JM, Douek DC (2008) HIV infection and the gastrointestinal immune system. Mucosal Immunol 1 (1): 23–30. 10.1038/mi.2007.1 PMC2777614 19079157

[44] Achkar JM, Lawn SD, Moosa MY, Wright CA, Kasprowicz VO (2011) Adjunctive tests for diagnosis of tuberculosis: serology, ELISPOT for site-specific lymphocytes, urinary lipoarabinomannan, string test, and fine needle aspiration. J Infect Dis 204 Suppl 4S1130–S1141. 21996695 10.1093/infdis/jir450 PMC3192548

